# Impacts of river fragmentation on limiting individual dietary specialization of Amazonian predatory fish

**DOI:** 10.7717/peerj.14266

**Published:** 2022-12-15

**Authors:** Jamerson Aguiar-Santos, Pieter deHart, Bruce Forsberg, Carlos Freitas

**Affiliations:** 1 Graduate Program in Ecology, National Institute of Amazonian Research, Manaus, Amazonas, Brazil; 2 Office of Graduate Studies, University of Wisconsin-Green Bay, Green Bay, WI, United States of America; 3 Department of Environmental Conservation, Vermont Agency of Natural Resources, Montpelier, VT, United States of America; 4 Department of Fishery Sciences, Federal University of Amazonas, Manaus, Amazonas, Brazil

**Keywords:** Intrapopulation niche, Individual niche, Trophic niche, Peacock bass, *Cichla temensis*, Habitat fragmentation

## Abstract

Individual dietary specialization is one of the factors that promotes variation in resource use at the individual level. Here we used stable isotope analysis of multiple tissues with different turnover rates to examine the degree of individual specialization in two sub-populations of the predator *Cichla temensis* inhabiting both fragmented and undammed rivers within the Uatumã River basin of the Amazon. Our results showed that the undammed river provides better conditions to promote individual dietary specialization than the fragmented river. This study contributes to the understanding of how specific life history characteristics of populations of generalist predators are impacted by fragmentation within megadiverse environments such as the Amazon basin.

## Introduction

Like other populations of predators, predatory fish, normally considered generalists, can have populations composed of both generalist and specialist individuals, that is, individuals of the same species can use resources in different ways (Bylak, 2018; Balme et al., 2020). Some studies have suggested that among-individual niche variation or individual dietary specialization is a common phenomenon that can affect the ecological dynamics of a population, contribute to niche variation within the population (Bolnick et al., 2003), and even alter community ecology more broadly. Araújo, Bolnick & Layman (2011) summarized the role of different mechanisms, such as interspecific competition and ecological opportunism, that can promote individual specialization in natural populations.

In response to interspecific competition, individual specialization may increase or decrease depending on the distribution of resources. If the competitor reduces the abundance of preferred resources, individuals may use alternative resources or change habitats depending on resource availability (Futuyma & Moreno, 1988; Bylak, 2018). In the tropics, ecological opportunity (high diversity of resources) may be more important than interspecific competition in promoting niche variation among individuals (Araújo & Costa-Pereira, 2013). Individual-level dietary specialization may be common in many species but has rarely been investigated in Amazonian predatory fish.

A fish can often use the food sources more profitably at different times (Gerking, 1994; Mortillaro et al., 2015). The speckled peacock bass, *Cichla temensis*, is an Amazonian diurnal predator able to feed on a variety of prey and take advantage of the most abundant prey (Aguiar-Santos et al., 2018; Hoeinghaus et al., 2006). This opportunistic behavior can be advantageous in the Amazonian environment, which provides a wide variety of food items for fish. However, the diversity and supply of food sources are subject to strong seasonal variations (Junk, Bayley & Sparks, 1989). In general, predatory fish can exploit a wide variety of prey species that become available during the hydrological cycle (Lowe-McConnell, 1999; Mérona & Rankin-de Mérona, 2004). For example, in the Amazon basin, the piranha *Serrassalmus gouldingi* begins ingesting fruits and seeds as they become available during the flood season, but fish fragments are the main source of food during the other hydrological periods (Prudente et al., 2016). A high degree of feeding plasticity is common among predatory species and may reflect opportunistic feeding choices at the individual level (Neves et al., 2021), but this dynamic may be altered by river fragmentation (Mérona & Vigouroux, 2006).

One of the largest drivers of river fragmentation is the increasing abundance of hydroelectric dams (Latrubesse et al., 2017). While dams have afforded society many benefits, regulated rivers are ecologically and physically different from undammed rivers (Collier, Webb & Schmidt, 1996). In the Amazon, specifically, the Balbina Hydroelectric Dam was constructed more than thirty years ago on the Uatumã River, and it has since caused several downstream impacts. Changes in predictability, amplitude, duration, and frequency of river flow have impacted the hydrology and floodplain ecosystems downstream which are adapted and dependent on seasonal variation in river level (Assahira et al., 2017; Rocha et al., 2020; Schöngart et al., 2021; Melack et al., 2021). Impacts on alluvial forests downstream from dams can reduce fish richness and abundance, which can alter overall fish diversity (Lobón-Cerviá et al., 2015). Besides the impacts of large hydroelectric dams, small hydropower plants (SHPs) also can cause substantial environmental impacts (Couto & Olden, 2018). SHPs may lead to the disappearance of fish species or the isolation of populations as occurs with large hydroelectric dams (Kukuła & Bylak, 2022). In the Amazon Basin, thousands of small dams are operating and acting as physical barriers to fish movement (Freitas et al., 2022). Blocking movement between upstream and downstream stretches, small dams can alter the composition and trophic structure of fish assemblages in Amazonian streams (Sousa et al., 2018). These changes in the fish assemblage in turn influence the prey-predator relationships, the fish prey composition, and overall resource availability for predatory fish.

Several approaches have been used to quantify the variation in resource use at the individual level (Bolnick et al., 2003), and stable isotope analysis has been shown to be a useful tool for inferring the resource niche-breadth of individuals over time (Lima et al., 2019). The use of different types of tissue of the same individual permits the assessment of dietary specialization on different time scales due to different rates of tissue incorporation of recent dietary inputs (Martínez del Rio et al., 2009). Quevedo, Svanbäck & Eklöv (2009) showed marked intrapopulation niche partitioning in a generalist predator using tissues formed at different times of trophic activity integration.

In this study, we investigated whether sub-populations of the predator *C. temensis* from two distinct fluvial environments differed in their degree of trophic resource specialization. Specifically, we used carbon and nitrogen stable isotope analysis (*δ*^13^C and *δ*^15^N, respectively) of prey fish and multiple tissues with different turnover rates (*i.e.,* muscle and caudal fin) in *C. temensis*, to examine the degree of individual trophic specialization in distinct *C. temensis* populations inhabiting a dammed and an undammed reach of the Uatumã River system, Amazon basin. In addition, we tested the influence of standard length and trophic position on individual specialization values of *C. temensis* individuals, using the *δ*^15^N levels in autotrophic energy sources to determine the trophic position of *Cichla*. We hypothesized that the undammed river, due to its greater environmental complexity and diversity of food sources, could offer better environmental conditions to promote individual specialization than the fragmented river.

## Material and Methods

### Study area

**Figure 1:**
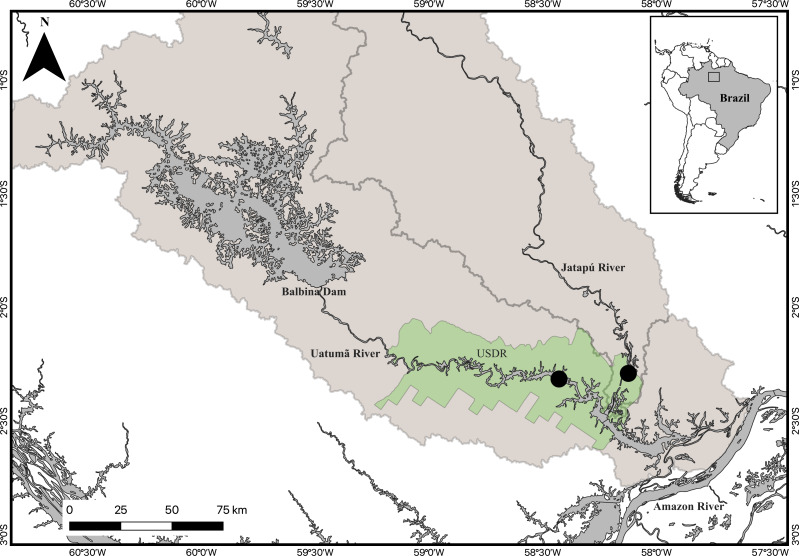
Map of the study area. The inserted map represents the location of the study area in Brazil. The Uatumã Sustainable Development Reserve (USDR) is shown with the green shaded color, including the locations of the sampled sites (filled circles) downstream from the Balbina dam, Amazonas, Brazil. The darker grey area represents the Balbina dam reservoir. Broader shaded area is the Uatumã River watershed.

Fieldwork was carried out at the Uatumã Sustainable Development Reserve (USDR) in the Brazilian State of Amazonas. The USDR is situated in the lower course of the Uatumã and Jatapú rivers (Fig. 1). The Uatumã River is regulated by the Balbina hydropower dam, which was built in the middle reach of the Uatumã River in 1987. Balbina Dam has a nominal capacity of 250 MW, but is highly inefficient, generating only 112.2 MW, on average (Fearnside, 2015). The environmental impacts found upstream of the dam were magnified by the formation of a huge reservoir. The downstream reach of the Uatumã also was impacted by massive tree mortality, decrease in the maximum water level, increase in the minimum water level, diversity loss, increased greenhouse gas emissions, and high methylmercury levels in aquatic biota (Kemenes, Forsberg & Melack, 2007; Kasper et al., 2014; Schöngart et al., 2021). In contrast, the Jatapú River is an undammed river. It is the main tributary of the Uatumã River with its confluence located approximately 228 km downstream from the Balbina dam (Schöngart et al., 2021). The Jatapú River originates in the Guiana Shield and drains areas with low population density, including several indigenous protected areas, with a low degree of land use change. Both the Uatumã and Jatapú are acid black water rivers with pH ranging between 4 and 5, low inorganic nutrient concentrations, and high levels of dissolved organic materials, mainly humic and fulvic acids (Junk et al., 2015).

### Ethical statement

All procedures performed in the study were approved by the Ethics Committee on Animal Use of the Federal University of Amazonas, Manaus, Brazil (Protocol No. 023/2019). The specimens were collected with authorization for activities with scientific purposes by license number 65955-1 SISBIO/IBAMA/MMA.

### Sample collection

Sampling was performed in October 2020, during the falling water period. Samples of *C. temensis* were collected in both the Uatumã and Jatapú Rivers approximately 25 km upstream from their confluence. All fish were caught with a fishing rod and artificial bait. After capture, the fish were euthanized through hypothermic treatment in an isothermal box with water and ice. Standard length (SL, cm) was recorded for each captured fish. Muscle tissue from the middorsal region was removed, stored in glass scintillation vials, and placed in a freezer until processing and analysis for stable isotopes. Caudal fin tissue with a slower turnover rate was also collected to infer individual specialization over a longer time period (Araújo, Bolnick & Layman, 2011). Tissue from the upper lobe of the caudal fin, including fin rays, was removed from each individual and stored in a 1.5 ml centrifuge tube with 70% alcohol solution. Since the isotopic values can be affected by the method of tissue preservation, the isotopic values were corrected using an equation for ethanol treatment provided by Planas et al. (2020) for finfish.

Since the diversity of food sources (prey fish) may be different between the rivers, the fish assemblage was sampled at each sampling site. Potential fish prey were collected using gillnets (2 m height ×15 m length) and mesh sizes varying from 30–120 mm. Gillnets were left fishing for 4 h in the morning (05:00–09:00 h) and 4 h in the evening (17:00–21:00 h). Dorsal muscle samples were collected from each individual and stored as described above. In addition, suspended particulate organic material (SPOM), predominantly phytoplankton, was collected at each sampling site and analyzed for stable isotopes to represent the autotrophic (base) energy source in the *C. temensis* food chain. These samples were collected using a 25 a µm plankton net dragged horizontally three times in the subsurface water, then stored in 500 ml bottles. The samples were collected on pre-combusted Whatman GF/F glass fiber filters using a vacuum filtration system under low pressure and stored until analysis.

### Sample processing

All biological samples were dried in an oven at 60 °C for 24 h. After desiccation, all samples were transported to the University of Wisconsin (Green Bay, WI, USA) for isotopic analysis. Sample preparation was performed as previously described in deHart, Powers & Hyzy (2016). All samples were cleaned using double-distilled water and freeze-dried before the subsampling procedure. Each sample was ground into a fine powder using a mortar and pestle. To obtain values for *δ*^13^C and *δ*^15^N, 1.0 ± 0.2 mg of this material was then subsampled into pre-weight tin capsules (Costech 5 × nine mm) and weighed using a Sartorius CPA2P microbalance. C and N isotopic composition were analyzed at the Central Appalachians Stable Isotope Facility in Frostburg, Maryland (USA), using a Carlo Erba NC2500 elemental analyzer interfaced with a Thermo Delta V+ isotope ratio mass spectrometer. Stable isotope ratios were expressed as *δ*^13^C or *δ*^15^N = ((*R*_sample_/ *R*_standard_) − 1) ×1,000, where R_sample_/R_standard_ are the ratios of ^13^C/^12^C and ^15^N/^14^N. Data were expressed using delta notation (*δ*) in parts per thousand (‰) with the reference material for *δ*^13^C being Vienna PeeDee Belemnite for *δ*^13^C and atmospheric air for *δ*^15^N. Measurement precision was estimated at ±0.11‰ and ± 0.12‰ for *δ*^15^N and *δ*^13^C, respectively. Lipid corrections of *δ*^13^C values were not considered appropriate because all fish samples displayed C/N ratios <3.5 (Post et al., 2007).

### Data analysis

The degree of individual specialization for both populations of *C. temensis* was measured using an index of within-individual component (WIC) to total niche width (TNW) for continuous data, for both *δ*^13^C and *δ*^15^N values from muscle tissue and caudal fin of each specimen. The TNW of a population is composed of the sum of its WIC and between individual component (BIC) (Roughgarden, 1972; Bolnick et al., 2003). Individual specialization is computed as the ratio between WIC and TNW. The WIC/TNW index compares the average individual’s niche to the population niche. This index ranges from zero when the population is composed of specialists that use a small subset of the population niche, to 1, when individuals are generalists and use the same resources as the population (Bolnick et al., 2002). To test the significance of the WIC/TNW index against the null hypothesis that individuals are all generalists, 10^3^ replicates were generated through a non-parametric Monte Carlo bootstrap procedure, which resulted in values referred to as *p* values (Zaccarelli, Bolnick & Mancinelli, 2013).

Prey fish were divided into four functional groups: carnivores, detritivores, herbivores and omnivores (Sleeg & Albert, 2018). The relative contributions of these prey groups for each individual of *C. temensis* were estimated using the *δ*^15^N and *δ*^13^C values of prey group muscle tissue and *C. temensis* muscle tissue and caudal fin with a Bayesian mixing model from the MixSIAR R package (Stock et al., 2018). Before applying the mixing model, we compared the variances of the caudal fin samples to account for the sample size differences through F-test. The individual identity was included in the models as a random effect. The fish prey groups are the same used by Aguiar-Santos et al. (2022) that were collected in the same areas and at the same time as individuals of *C. temensis*. From the outputs of the MixSIAR models, we calculated the degree of specialization for each individual through the individual specialization (IS) index. The IS index quantifies the mean proportional similarity (PS_i_) between the individual diet and the population diet (Bolnick et al., 2002). This index spans from 0, when individuals are specialists, to 1, when all individuals are generalists. That is, when there is complete overlap between the diet of the individual and the population, there is no individual specialization. We used Monte Carlo bootstrap permutations with 10,000 replicates to test whether observed IS values differed from a random distribution of values subsampled from the population. These analyses were performed using the RInSp package (Zaccarelli, Bolnick & Mancinelli, 2013).

To identify differences in the degree of individual specialization between populations, and to test whether the standard length and the trophic position influence the degree of individual specialization, we perform a generalized linear model (GLM) using beta family distribution in the betareg package (Cribari-Neto & Zeileis, 2010). This distribution is used when proportional data assume values in the standard unit interval (0,1). Nitrogen isotopic values for SPOM (phytoplankton, baseline) and *Cichla* (consumer) were used to estimate the trophic positions of *C. temensis* individuals following the equation: Trophic Position (TP) = [1 + *δ*^15^N_consumer_ − *δ*^15^N_baseline_)/3.04‰], where 1 is the trophic level of the baseline organism (phytoplankton) and 3.04‰ is the estimated fraction per trophic level (Post, 2002; Bastos et al., 2017). We only used isotopic information from fish muscle tissue for these analyses because it had a similar number of replicates per population.

Finally, we performed an analysis of variance (ANOVA) to compare the trophic position of *C. temensis* individuals in both populations. We also calculated the proportion of the trophic groups in each sampling site to analyze the influence of the potential prey fish assemblage on the trophic position of *C. temensis* populations. The percentage of each trophic group (Carnivores, Detritivores, Herbivores, Omnivores) was calculated by dividing the number of species on each trophic group in each sampling site by the total number of species.

## Results

In total, we sampled 48 adult individuals of *C. temensis* (Table 1). Eight caudal fin samples were lost from the Jatapú collection. Individual isotopic variation in *C. temensis* was larger for energy source use (*δ*^13^C) than the trophic level (*δ*^15^N) for both populations (Table 2). The WIC component accounted for 81.22% and 46% of the TNW variation in *δ*^13^C and *δ*^15^N of the Uatumã River *C. temensis* population, and 89.1% and 64.28% of the TNW variation in *δ*^13^C and *δ*^15^N of Jatapú River *C. temensis* population. The individual specialization index WIC/TNW showed values close to 1 for *δ*^13^C and low values for *δ*^15^N for both populations. The simulations generated by the Monte Carlos bootstrap procedure did not indicate the presence of specialist individuals (Table 2).

**Table 1:** Mean (±SD) *δ*^13^C and *δ*^13^N values of *C. temensis* populations.

Population/tissue	*n*	*δ*^13^C	*δ*^15^N
*Uatumã River*			
Muscle tissue	27	−35.95 (1.41)	14.43 (0.82)
Caudal fin	27	−30.95 (1.19)	14.15 (0.92
*Jatapú River*			
Muscle tissue	21	−35.51 (1.22)	13.55 (0.82)
Caudal fin	13	−30.36 (0.99)	11.95 (0.60)

**Notes.**

n Number of individuals

The isotopic values are expressed in ‰.

**Table 2:** Individual specialization metrics for *C. temensis* populations.

Population	TNW	WIC	BIC	WIC/TNW	*p* value
*δ*^13^C					
Uatumã	7.88	6.40	1.48	0.81	0.99
Jatapú	8.44	7.52	0.92	0.89	0.99
*δ*^15^N					
Uatumã	0.75	0.35	0.40	0.46	0.32
Jatapú	1.12	0.72	0.39	0.64	0.81

**Notes.**

TNW Total niche width WIC Within-individual component BIC Between-individual component

**Figure 2:**
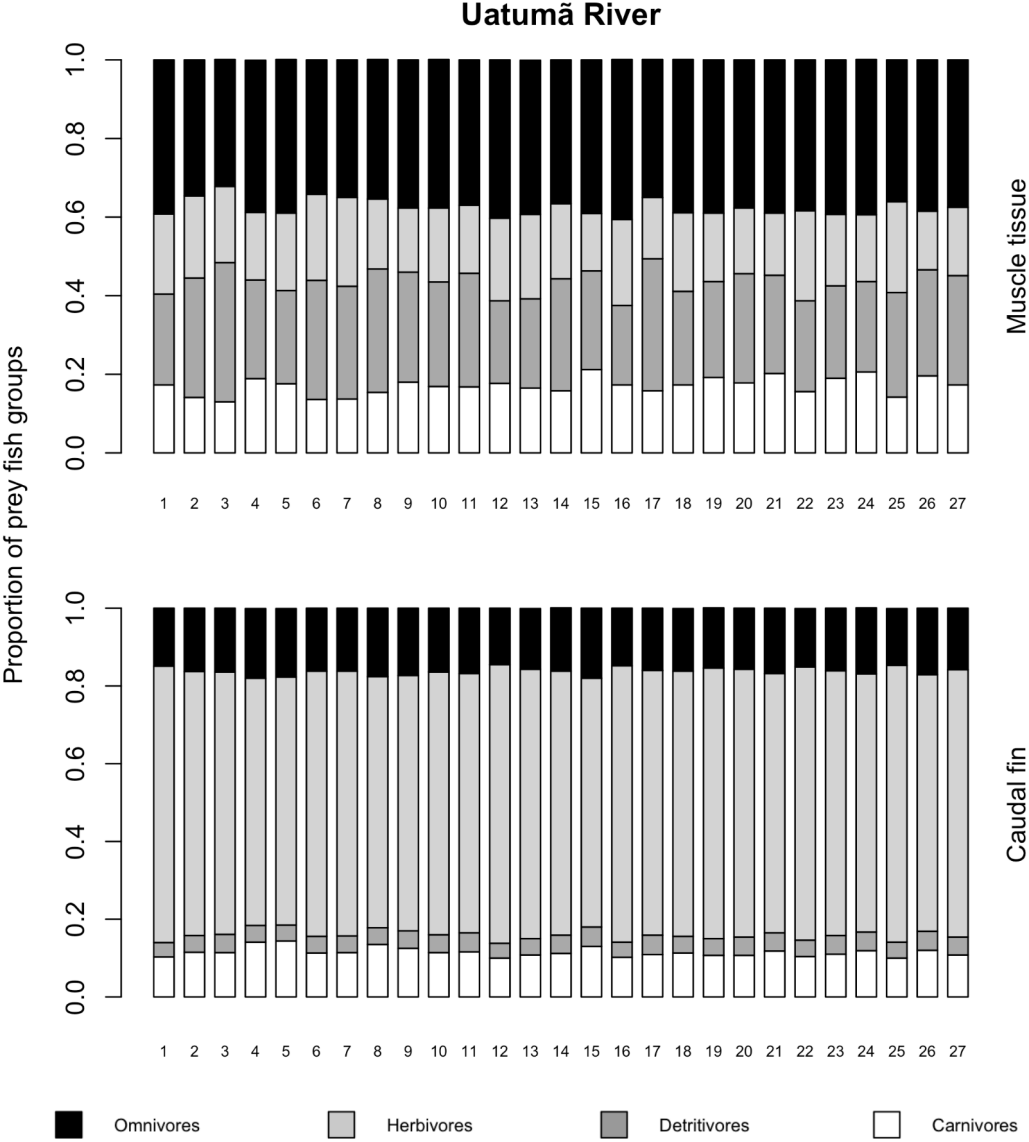
Contribution of fish prey groups to the diet of *C. temensis* individuals obtained from each tissue type in the Uatumã River. The lower axis is individual fish.

**Figure 3:**
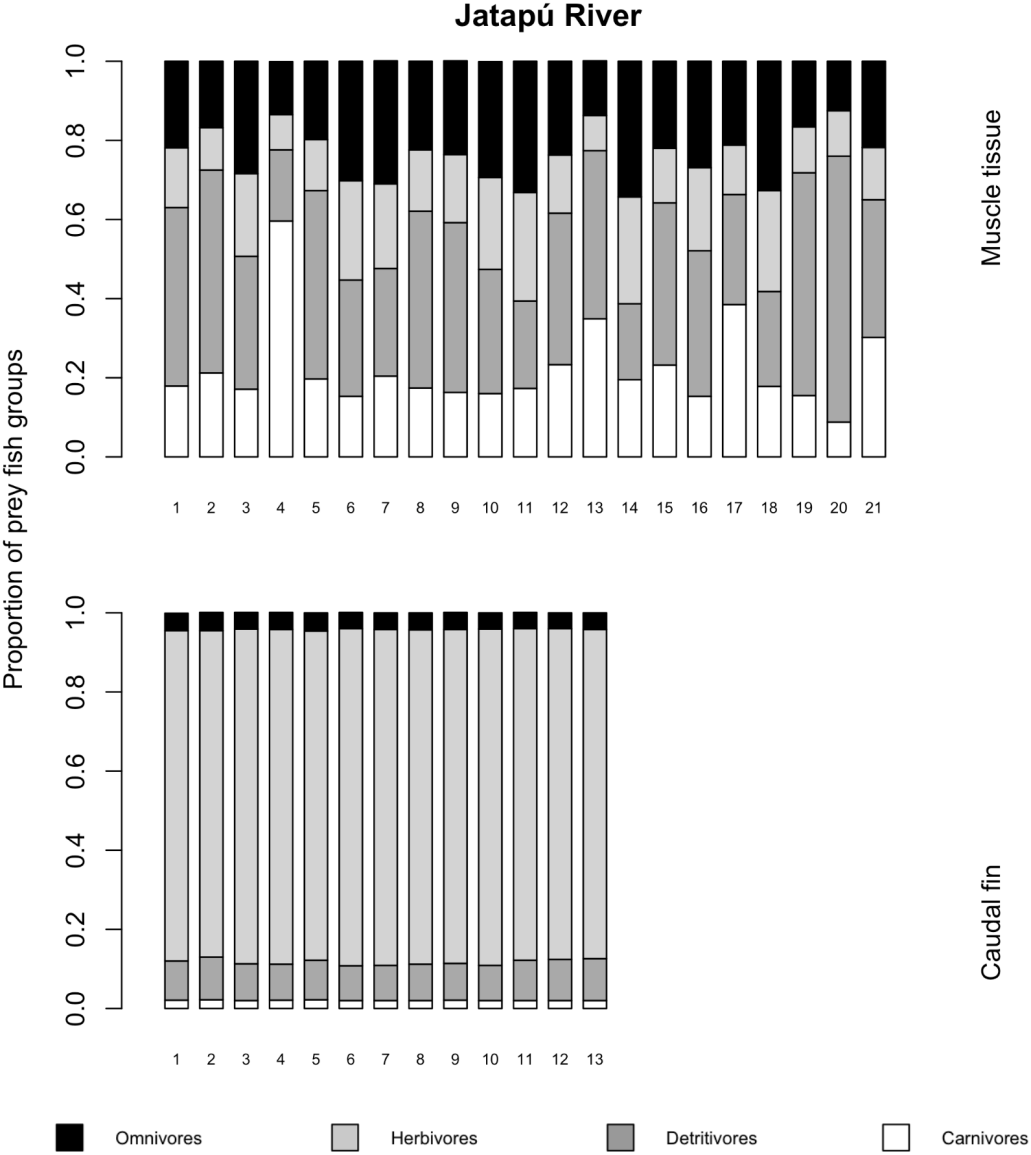
Contribution of fish prey groups to the diet of *C. temensis* individuals obtained from each tissue type in the Jatapú River. The lower axis is individual fish.

**Figure 4:**
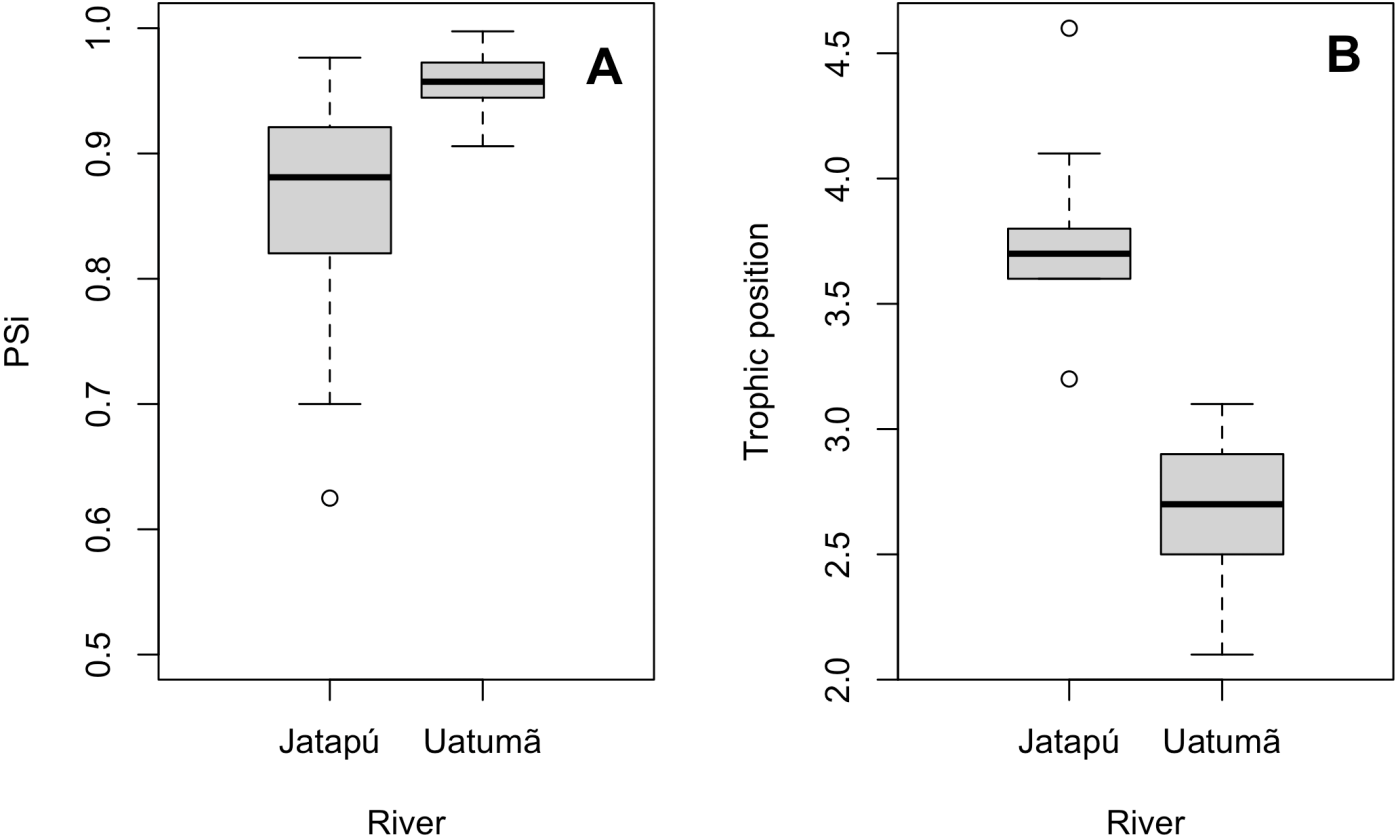
Comparison of the proportional similarity values and the trophic position values by population. (A) Boxplot of proportional similarity values (PS_i_) by rivers and (B) boxplot of trophic position of *C. temensis* by rivers. The Uatumã River is the dammed river and the Jatapú River is the undammed river.

**Figure 5:**
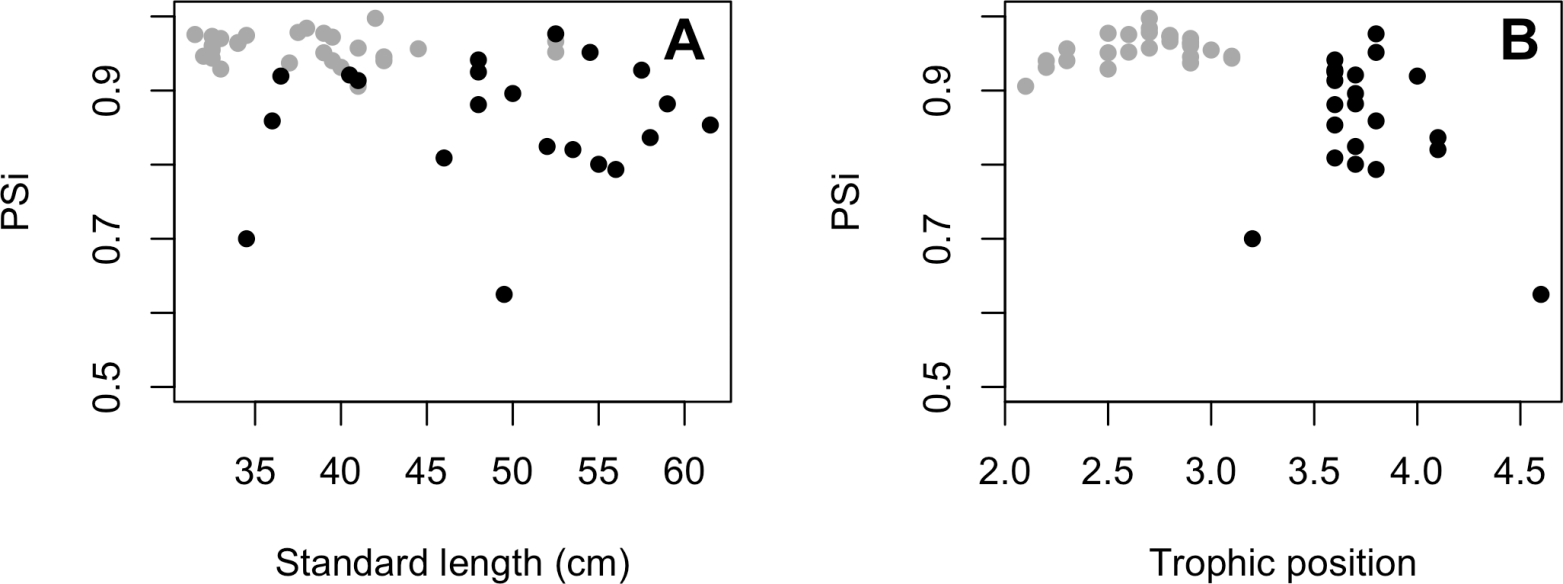
(A) Relationship between proportional similarity values (PS_i_) and standard length. (B) Proportional similarity values (PS_i_) and trophic position of *C. temensis*. Each point represents an individual of *C. temensis* from the dammed river (gray circle) and the undammed river (black circle). There is no effect of standard length or trophic position on PS_i_ values.

The variances of *δ*^13^C and *δ*^15^N of the caudal fin are homogeneous (Table S1). MixSIAR outputs for muscle samples indicated that omnivores and detritivores were the most consumed fish prey groups for both *C. temensis* populations (Figs. 2 and 3). The results obtained from caudal fin showed that the herbivores were the most important prey fish group for both populations (Figs. 2 and 3), and the IS values calculated from dietary estimates for both tissues were high (Table 3). The Ps_i_ values differed between the *C. temensis* populations (Table 4) with lower values in the Jatapú River *C. temensis* population as compared to the Uatumã River *C. temensis* population (Fig. 4A). However, neither the standard length nor the trophic position influenced the PS_i_ values (Table 4; Fig. 5).

**Table 3:** Mean estimated contribution and standard deviation of fish prey groups and individual specialization index (IS) index for *C. temensis* populations.

	Carnivores	Detritivores	Herbivores	Omnivores	IS	*p*-values
*Uatumã River*						
Muscle tissue	0.17 (0.009–0.36)	0.26 (0.13–0.38)	0.19 (0.01–0.38)	0.38 (0.01–0.75)	0.96	1
Caudal fin	0.11 (0.007–0.25)	0.04 (0.002–0.12)	0.68 (0.46–0.86)	0.16 (0.005–0.46)	0.98	1
*Jatapú River*						
Muscle tissue	0.21 (0.09–0.34)	0.38 (0.26–0.50)	0.17 (0.01–0.32)	0.24 0.01–0.55)	0.86	1
Caudal fin	0.02 (0.001–0.06)	0.09 (0.006–0.22)	0.84 (0.72–0.95)	0.04 (0.001–0.14)	0.99	0.34

**Table 4:** Results of the Generalized Linear Model (GLM) examining variation of the trophic position, standard length and site on proportional similarity values (PSi) of the *C. temensis* individuals.

Predictor variable	Estimate (*SE*)	*z*	*p*
Intercept	2.92 (1.32)	2.21	**0.03**
Trophic position	−0.304 (0.31)	−0.98	0.32
Standard length	0.002 (0.01)	0.16	0.87
River (Uatumã)	0.7675 (0.40)	1.91	**0.05**

**Notes.**

SE Standard error

We modeled PSi values from muscle tissue of *C. temensis* individuals with beta regression (R function betareg). Model: PSi ∼Trophic position + Standard length + River. The significant (*p* < 0.05) effects are given in bold.

**Figure 6:**
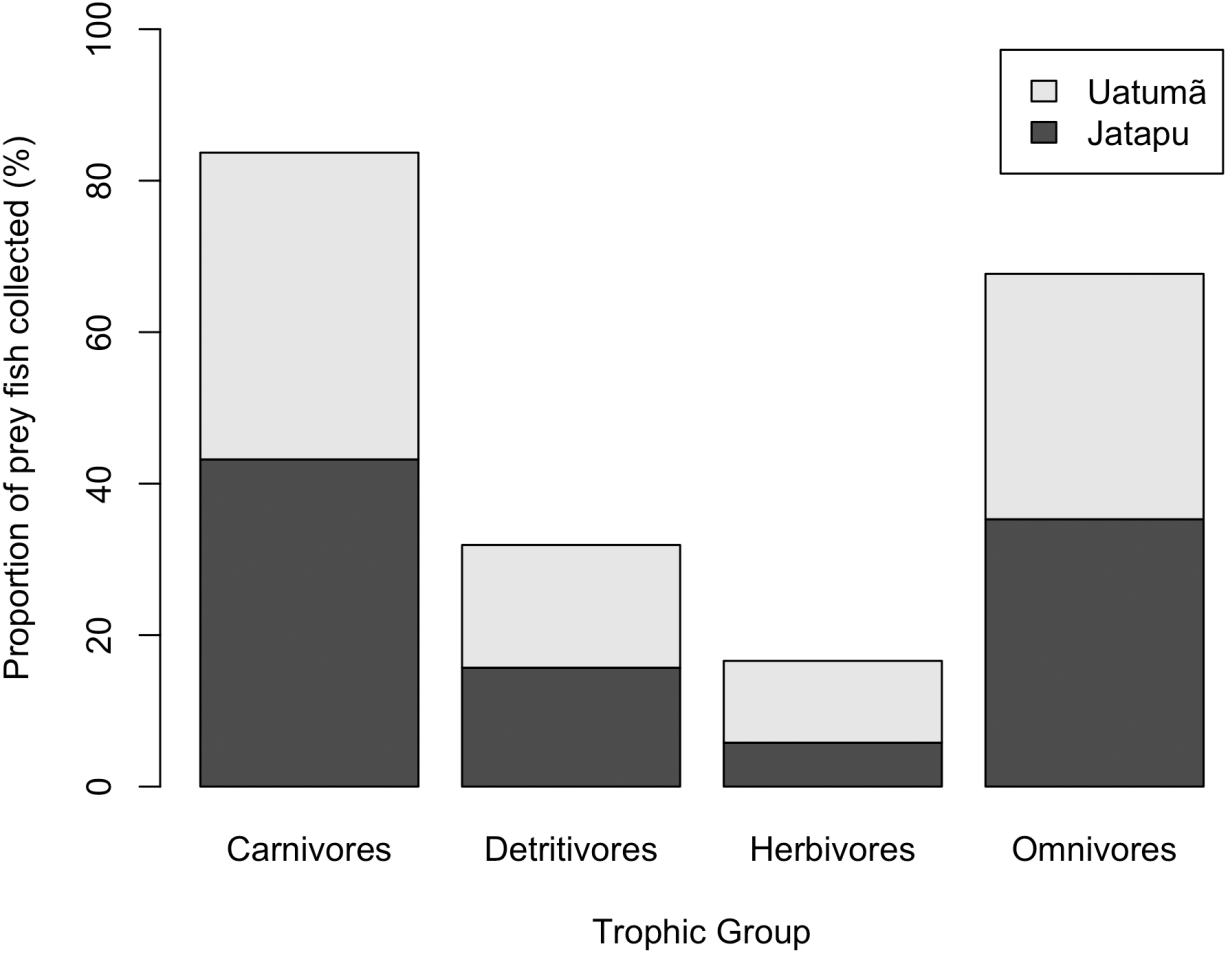
The proportion of potential prey trophic groups collected in the dammed river: Uatumã River, and in the undammed river: Jatapú River.

The Jatapú River *C. temensis* population has higher trophic position values than the Uatumã River population (ANOVA: *F* = 179; *df* = 1,46; *p* < 0.05, Fig. 4B). Sixty prey species were recorded in total, consisting of 23 species recorded only in the Jatapú River, nine species recorded only in the Uatumã River, and 28 in both rivers. The trophic composition of the potential prey fish in the Jatapú River was comprised of 43.2.% carnivores, 35.3% omnivores, 15.7% detritivores, and 5.8% herbivores. In the Uatumã, the proportion of trophic groups was 40.5% carnivores, 32.4% omnivores, 16.2% detritivores, and 10.8% herbivores (Fig. 6).

## Discussion

Individual dietary specialization in typically generalist populations is a widespread phenomenon but has rarely been investigated in Amazonian predatory fish (Bolnick et al., 2003). Our study used stable isotope analysis of multiple tissues with different turnover rates to analyze the degree of trophic resource specialization of *C. temensis* populations inhabiting a fragmented and an undammed reach of the Uatumã River system, Amazon basin. Our results showed that the undammed river reach may provide better conditions to promote individual specialization than the fragmented reach we considered. Specifically, while our analysis of WIC/TNW values and the high IS values calculated from the dietary estimates for both tissues both suggest a low proportion of specialist individuals in populations of both rivers, the overall differences in PS_i_ values between the two river populations suggest a higher degree of individual specialization of *C. temensis* in the Jatapú river as compared to the Uatumã river. Additionally, the prey fish assemblage did vary between the two rivers, which affected the trophic position of *C. temensis* and may have influenced the degree of specialization. These results suggest that the *C. temensis* population of the Jatapú river is less generalist than the population of the Uatumã river.

In natural habitats, individuals of the *C. temensis* are opportunist strict piscivores that consume a variety of fish prey (Aguiar-Santos et al., 2018). In these environments, the feeding ecology of the organisms is influenced by seasonal and predictable variations in river level which affect the availability of foraging areas and food resources (Junk, Bayley & Sparks, 1989). This dynamic affects the abundance and composition of the fish assemblage and consequently influences prey composition (Silva et al., 2021). As with other populations of predatory fish in the Amazon basin, some individuals may show some trophic specialization. In the Rio Negro, some individuals of the predator *Acestrorhynchus falcirostris* show a diet specialized in the consumption of seasonally abundant prey, while others are more generalist, consuming prey that occurs throughout the year (Lubich et al., 2024). Thus, variable prey availability associated with phenotypic characteristics may drive prey capture, leading to differing degrees of individual specialization (Cardoso et al., 2019).

Habitat fragmentation by hydroelectric dams causes multiple impacts on the structure, dynamics, history, and functioning of freshwater ecosystems (Pelicice et al., 2021). For example, the fragmentation of the Madeira River has modified the food spectrum of piscivores mainly changes in piscivorous abundance, diet composition, niche breadth, and resource partitioning among piscivorous fish (Lonardoni et al., 2021). The regular and predictable variation in water level in the Uatumã River was altered by the construction of the Balbina hydroelectric plant; these changes in the fluvial flood pulse modified the composition, distribution and structure of the biological communities (Arantes et al., 2019; Schöngart et al., 2021). Fish populations lost important food sources due to the loss of floodplain forest (*igapó* forest), which then leads to a reduction in the abundance of potential prey associated with these environments. Thus, in fragmented habitats such as those that have been dammed, individuals need to expand their diet to include alternative food items to meet their dietary requirements. Besides losses of the ecologically unique and important *igapó* forest by the damming, the Uatumã River has also been altered by human settlements with different land cover types (Resen de et al., 2019). The combination of all these factors contributes to the diet generalization of individuals of the *C. temensis* population in the Uatumã River.

Ecological opportunity may favor specialization in populations that have access to a greater diversity of prey (Balme et al., 2020). In the Negro River basin, *C. temensis* adult individuals showed a narrow trophic niche with a highly specialized diet based on prey fish consumption (Aguiar-Santos et al., 2018). In the dam-impacted region of the Uatumã River, the *C. temensis* population had a broader trophic niche and higher exploitation of carbon sources than the population of the undammed river within the same watershed. While in the undammed river, individual *C. temensis* exploited prey at higher trophic levels (Aguiar-Santos et al., 2022). Non-fragmented habitats have better conditions to promote individual specialization because of the greater availability, diversity, and seasonal fluctuations of prey fish (Araújo, Bolnick & Layman, 2011). Thus, the difference in the trophic structure of potential prey assemblages drives differences in the degree of trophic specialization and trophic position of *C. temensis* populations.

Stable isotopes have been used to quantify individual specialization through studies which examine multiple types of tissues with different metabolic rates from the same individual to infer dietary habits in different periods (Dalerum & Angerbjörn, 2005). There are very few studies reporting isotopic turnover rates of caudal fin for freshwater fishes. The few works that exist report that fin samples exhibit slow isotopic turnover in relation to liver and blood samples for cartilaginous fish and fast turnover (∼1 month) in teleost fish (German & Miles, 2010; Malpica-Cruz et al., 2012). The isotopic signature of muscle tissue of fish species reflects the diet of approximately 3-4 months ago but there is no consensus on the replacement rate of white muscle tissue in freshwater fish. Some authors reported 13.9 days to 85 days for tissues to equilibrate with dietary isotopic signatures (Sacramento, Manetta & Benedito, 2016). The time period and region examined in this study correspond to the peak flood and early falling water period; the period corresponding to the reproductive peak of *C. temensis* (Campos, Freitas & Amadio, 2015). During this period, individuals form pairs, prepare nests, and perform mouthbrooding; the parents take turns caring for the offspring and show characteristics of guardians and territoriality behavior. Throughout this time, feeding activity is reduced. Individuals capture their prey by ambush and/or pursuit, targeting prey that approaches the nests (Jepsen, Winemiller & Taphorn, 1997). These feeding behaviors are all unique to the reproductive period of a generalist predator, and so it is unsurprising that we did not detect the presence of specialist individuals in both populations during that window of time.

Outside the reproductive period, in the Cinaruco River –Venezuela, large individuals of *C. temensis* can move over moderate distances (∼21 km) (Hoeinghaus et al., 2003). This behavior can be associated with the seasonal exploiting of large schools of *Semaprochilodus kneri* that can provide large piscivores with nutrients, fueling high rates of growth, fecundity, and recruitment (Winemiller & Jepsen, 2002; Hoeinghaus et al., 2006). This seasonal contribution of prey to the diet of some individuals can lead to increased variation in diet among individuals, favoring an increase in populational trophic niche width (Araújo, Bolnick & Layman, 2011). Therefore, ecological opportunity and competition for valuable resources among individuals in the population can promote individual specialization.

On the other hand, interspecific competition weakens individual specialization in species-rich communities (Araújo, Bolnick & Layman, 2011). The presence of a competitor species increases the pressure on food resources and forces niche differentiation between the competitors. Amazonian aquatic environments, in particular, have high species richness and resources (Reis et al., 2016); it would not be metabolically beneficial for predators in megadiverse environments, living in conditions characterized by seasonal changes such as those found in the Amazon to specialize on specific prey species, because they need to modify their diet to ingest the food resources that are available at different times of the year (Lowe-McConnell, 1999). In periods of food scarcity, there is an increase in population trophic niche width and a greater overlap of individual niches with the population niche. In the central Amazon floodplain, four out of six piscivores increased their trophic niche breadth in the low water season, replacing part of their fish diet with shrimp or even vegetable matter to reduce interspecific competition (Mérona & Rankin-de Mérona, 2004). In doing so, the interspecific competition in this environment changes and can lead predator fish to behave more like a trophic generalist and yield lower abundance and densities of specialist individuals in their populations. Even still, the high diversity of food resources associated with the seasonal variation in water level remains an environmental opportunity and can provide conditions for individuals to exhibit more specialized diets.

The use of multiple stable isotope analyses in distinct tissues with different turnover rates allowed us to compare the degree of individual specialization in two sub-populations of an Amazonian predator fish in dammed and undammed river reaches. However, some caveats must be considered in this analysis. The inferences derived here from a single field collection, integrating feeding patterns over a limited part of the annual flood cycle (peak flood through early falling water) in two reaches of the same river system may be strong in this specific spatial and temporal context, but caution must be used in extrapolating these results to other rivers systems and hydrological periods. The hydrological complexity of the river reaches sampled in this study also makes it difficult to evaluate the specific impacts of impoundment on these systems. A large decline in the magnitude of the flood pulse and major impacts on alluvial flora and fauna were encountered along the first 50 km downstream from Balbina dam, but it is not clear whether similar impacts have occurred at our collection sites ∼150 km downstream (Fig. 1). Both sites are much closer to the confluence of the Amazon River than to the dam and backwater effects from the Amazon are also expected to have strong effects on the hydrological dynamics of the Uatumã and associated tributaries like the Jatapú (Meade et al., 1991). Additional investigations will be required before we can separate the influence of these two opposing hydrological impacts and their effects on the aquatic food webs in these tributaries. If discrete effects of impoundment on the Uatumã can be demonstrated, it may still be difficult to isolate its effects on predator food webs due to fish migrations. *C. temensis* is a relatively sedentary predator and is unlikely to migrate between our two sample sites (Hoeinghaus et al., 2003). However, some prey species migrate much longer distances and could carry the impacts of impoundment, reflected in their relative abundances, from the Uatumã to the undammed Jatapú River. These migration patters will need to be considered in future studies. The analysis of stomach contents in predators and prey samples could improve the understanding of the trophic relationships of these populations.

## Conclusions

In summary, although no specialist individuals were found in either population, our results suggest that the *C. temensis* population from the undammed river was less generalist than the *C. temensis* population from the dammed river. The undammed ecosystems offered better environmental conditions to promote trophic specialization of predator populations. Undammed rivers preserve characteristics that maintain the complexity and connectivity of riverine habitats and create ideal conditions for the maintenance of biological communities. These results contribute to the understanding of how populations of generalist predators are composed in megadiverse environments such as the Amazon River system. They also provide insight into the consequences of river fragmentation due to damming on individual niche variation in predatory fish. Future studies should consider the effect of seasonal variations in river level on the persistence of specialist individuals in predatory fish populations in Amazonian rivers.

##  Supplemental Information

10.7717/peerj.14266/supp-1 Supplemental Information 1 *Cichla temensis* raw dataset: isotopic values of the muscle and fin tissues Information about individual, site, standard length, total weight, carbon isotopic value, nitrogen isotopic value for both muscle and fin tissues.

10.7717/peerj.14266/supp-2 Supplemental Information 2 *Cichla temensis* raw dataset: Trophic position Information about individual, site, standard length, total weight, carbon and nitrogen isotopic values, baseline isotopic value, trophic position and PS_i_ values.

10.7717/peerj.14266/supp-3 Supplemental Information 3 Results of F-test testing the hypothesis of variances homogeneity

## Additional Information and Declarations

### Data Availability

The following information was supplied regarding data availability: The raw measurements are available in the Supplementary Files.
