# Supplementary material for: Impacts of river fragmentation on limiting individual dietary specialization of Amazonian predatory fish
Source: PeerJ. 2022 Dec 15;10:e14266. doi: 10.7717/peerj.14266 (PMC9760019; doi:10.7717/peerj.14266)
Supplement: Supplemental Information 3 [file peerj-10-14266-s003.docx]

Supplemental Table 1

**Impacts of river fragmentation on limiting individual dietary specialization of Amazonian predatory fish**

Jamerson Aguiar Santos, Pieter A. P. deHart, Bruce R. Forsberg, Carlos E. C. Freitas

**Table 1.** Results of F-test testing the hypothesis of variances homogeneity. Dependent variables: caudal fin δ^13^C values, and factor: *Cichla temensis* populations.

|  | Df | F | P-value |
| --- | --- | --- | --- |
| Jatapú caudal fin | 12 | 0.69055 | 0.5088 |
| Uatumã caudal fin | 26 |  |  |

**Table 2.** Results of F-test testing the hypothesis of variances homogeneity. Dependent variables: caudal fin δ^15^N values, and factor: *Cichla temensis* populations.

|  | Df | F | P-value |
| --- | --- | --- | --- |
| Jatapú caudal fin | 12 | 0.41974 | 0.1169 |
| Uatumã caudal fin | 26 |  |  |
